# Effect of adipose tissue-derived stem cells therapy on clinical response in patients with primary Sjogren’s syndrome

**DOI:** 10.1038/s41598-023-40802-5

**Published:** 2023-08-19

**Authors:** Fangfang Li, Junhui Lu, Xinlian Shi, Dongya Li, Tingting Zhou, Tianqi Jiang, Shengming Wang

**Affiliations:** 1 Department of Ophthalmology, The Affiliated Huai’an Hospital of Xuzhou Medical University, Huai’an City, China; 2 Department of Rheumatology, The Affiliated Huai’an Hospital of Xuzhou Medical University, Huai’an City, China; 3 Department of Stomatology, The Affiliated Huai’an Hospital of Xuzhou Medical University, Huai’an City, China

**Keywords:** Stem cells, Diseases, Rheumatology, Dentistry, Disease prevention, Quality of life, Therapeutics

## Abstract

The purpose of this trial was to clinically assess the effect and safety of Adipose Tissue-derived Stem Cells (ADSCs) treatment on primary Sjogren’s Syndrome (pSS). In this 6-month randomized, triple-blind, placebo-controlled clinical trial, pSS patients were randomly assigned to two groups. After demographic characteristics and clinical examination were achieved, local injection of ADSCs into bilateral glands was performed with patients in ADSCs group (n = 35) and placebo solution was used for another group (n = 39) at three time points. Patients were followed up at 1-, 3- and 6-month. At each visit, studies of clinical and laboratory outcomes, as well as subjective symptoms, were conducted. A total of 74 subjects who met the including criteria were allocated in two groups and eventually 64 subjects (86.5%) completed the treatments and the follow-up assessments. Secretion of salivary and lachrymal glands were significantly improved in 3-month (*P* < 0.05). A great improvement of European League Against Rheumatism Sjögren’s Syndrome Disease Activity Index (ESSDAI) was found after ADSCs treatment with intergroup comparison from baseline to follow-up (*P* < 0.05). There is also a significant difference of European Alliance of Associations for Rheumatology SS Patient Reported Index (ESSPRI) between the two groups in the follow-up (*P* < 0.05). A significant abatement of IgG, IgM, C3, C4 and ESR between two groups was observed in part of follow-up time points (*P* < 0.05). The ADSCs therapy can provide relief of oral and eye’s dryness in our trial in a short time and has potential improvement of subjective and systemic syndromes of pSS.

## Introduction

Sjogren’s syndrome (SS) is a systemic autoimmune chronic disease characterized by marked exocrine glandular dysfunction such as salivary and lachrymal glands, presenting a persistent dryness of mouth and eyes^1^. In some areas like China, the prevalence can reach as high as 5.6%, with a female-to-male ratio of 9:1 due to an association with hormonal changes^2^. SS process can also extend to several other organs and tissues including lungs, kidneys, blood vessels, skin, and nervous system, which may impair patients’ quality of life. pSS occurs in the absence of an associated systemic autoimmune disease, whereas secondary SS (sSS) occurs secondary to other autoimmune disorder, such as systemic lupus erythematosus, rheumatoid arthritis, interstitial lung disease or interstitial nephritis^3^. As a rule, sSS is an accompanying disease while the main autoimmune disease is the determinant of treatment decision. Consequently, this trial focuses on treatment outcomes in pSS.

pSS has a very heterogeneous clinical presentations and the etiology has not been fully elucidated. Infiltration of a large number of lymphocytes and expression of various cytokines in the exocrine glands were detected in animal models^4^. Therefore, the primary of therapy are limited to palliation of symptoms and prevention of complications. Systemic treatment is often ineffective and can result in major side effects. At present, local treatments such as artificial tears or oral sprays rely on patients’ compliance. The secretagogues including pilocarpine and cevimeline have been specifically approved for the treatment of xerostomia^5^. In recent reports, the properties of different monoclonal antibodies against B and T cells have been investigated widely^6^. However, these marketed medicines were reported to potentially induce lymphoma-gastrointestinal symptoms or cardiovascular and pulmonary side effects. They are thus contraindicated in certain patients^7^. Despite a multiplicity of approaches, no gold standard has been established and novel, well-studied and appropriate methods are needed.

In recent decades, a wide range of animal and clinic-based methods have emerged as novel therapeutic methods, with evidence showing that the transplantation of ADSCs may benefit in treating autoimmune disease^8^. Koichiro et al. recently conducted a study of regenerative therapy for rheumatoid arthritis by transferring of ADSCs in an animal model^9^. ADSCs, differentiated mesenchymal stem cells, show a great ability of multi-lineage proliferation, anti-inflammation, immunomodulation and self-renew. Some studies had suggested that ADSCs may represent an alternative therapeutic option for systemic lupus erythematosus and systemic sclerosis patients^10–12^. ADSCs could be of the most promising cell types because they are cultured from a relatively small amount of adipose tissue and the number of administration can be well controlled^13^.

According to our literature research, although many in vitro and in vivo studies have investigated the effects of ADSCs on exocrine gland function and autoimmune disease, to date, the effectiveness of ADSCs for patients with pSS in a clinical setting remains inadequate and inconclusive. With this background, the main objective of this trial is to evaluate the safety and potential efficacy of ADSCs on pSS patients, by assessing subjective symptoms, clinical and laboratory outcomes.

## Methods

### Ethical statement

This trial was conducted in full accordance with the 2010 CONSORT guidelines of the World Medical Association Declaration of Helsinki. The study protocol was approved by the Ethic Committee of the the Affiliated Huai'an Hospital of Xuzhou Medical University (institutional approval number: HEYLL2020325). It was registered at Chinese Clinical Trial Registry (Registration umber: ChiCTR2000033420, http://www.chictr.org.cn/, Date of first registration: 31/5/2020). A written informed consent was provided to each subject prior to the treatments.

### Participants

Patients were consecutively recruited referred to Department of Rheumatology, Stomatology and Ophthalmology, Affiliated Huai'an Hospital of Xuzhou Medical University. Eligible patients were diagnosed as having pSS according to the American College of Rheumatology (ACR) Diagnostic Criteria for Sjögren’s Syndrome. At least two syndromes with following three objective features will meet the criteria: (1)patients are newly diagnosed with pSS and have not received previous treatments; (2) Positive serum anti-SSA (Ro) and/or anti-SSB (La); (3) Labial salivary gland biopsy exhibiting focal lymphocytic infiltration with a focus score ≥ 1 focus/4 mm^2^; (4) Keratoconjunctivitis sicca with ocular staining score ≥ 3.

The exclusion criteria for all patients were: (1) not currently using daily eye drops for glaucoma, and has not had corneal surgery or cosmetic eyelid surgery in the last 5 years; (2) any of the following conditions: history of head and neck radiation treatment, hepatitis C infection, acquired immunodeficiency syndrome, sarcoidosis, amyloidosis, graft versus host disease, IgG4-related disease; (3) other systemic conditions that could affect the progression of pSS, such as Systemic lupus erythematosus and Rheumatoid arthritis; (4) a history disease of chronic obstructive sialadenitis disease; (5) status of pregnancy or lactation.

Patients were interviewed to provide of informations including their socio-demographic characteristics, life-style factors, compliance to drugs and self-care knowledge at study entry. Clinical assessments of disease activity were also performed at the first screening.

### Randomization and masking

Patients were assigned to two groups via a computer-generated randomization schedule. The ADSCs Group was injected with ADSCs while Placebo Group was received 0.9% saline solution administration. Concealed allocated codes were kept in signed and sealed envelopes. An external investigator performed the randomization. Investigators, patients and investigators were masked to group assignment. Blinding was revealed after the treatments were performed and the data were analysed.

### Sample size analysis

Sample size analysis using G Power version3.1.9.2 was determined with Walters’s method by considering two groups of subjects, an effect size of 0.30 and a standard deviation of 1.7 by a previous randomized controlled clinical study. With an assumption of normal sample distribution, we set power of 0.8 and an error of *α* = 0.05. Adding 10% with the compensation of drop-out during the trial period, we set an increase of 20%. The final sample including 26 subjects in each group were determined, so that a power of 0.8 was obtained.

### The preparation of ADSCs

The drug in this trial was a solution of ADSCs. Adipose tissues were harvested with liposuction of abdomen from a healthy donor. They are washed in phosphate-buffered saline (PBS) and digested of fat aspirates with 0.075% collagenase. These stem cells were then packed in dimethyl sulfoxide and human platelet lysate and 1% antibiotics at a culture condition of 37 °C and 5% CO_2_. The surface markers of CD73, CD90 and CD105 were identified by flow cytometry analysis, as well as negative for expressing CD14, CD11b, CD45, CD19, CD79 and HLA-DR. Tumorigenicity study, sterility test and negative test for Mycoplasma were performed before use.

### Procedures

The final therapeutic drug solution was composed of 5 million cells per milliliter of solution. After disinfecting the surface projection of bilateral parotid glands with Iodophor and draping in a sterile fashion, the injection site was anesthetized with 1% lidocaine. A dose of 1 ml ADSCs solution was diluted in 5 ml 0.9% saline solution. The final stem cell solution was injected into bilateral glands with 0.05 ml ADSCs and saline mixed-solution (5 × 10^4^ cells) per kilogram of the patient’s weight by a 5 ml-syringe and 0.45 mm- needle under ultrasonic guidance. The dosage and interval of ADSCs was injected according to our previous researches. Patients in Placebo Group were injected with 0.9% saline solution as forementioned proportion. Backflow should be performed against the blood to the syringe before injection. Patients were allowed to leave after resting for 15 min. The second injection was performed in bilateral glands in the second and fourth week under similar conditions. All subjects in this trial were reinforced with hygiene instructions and without any medical treatment. In this trial, the patients were clinical evaluated at four follow-up time points: before the intervention, 1-month, 3-month and 6-month after the third injection. During the follow-up, patients could withdraw in any time. Patients without a compliance rate more than two times were required to withdraw after approval from the principal investigator.

### Outcome measures

#### Sialometry

Patients were asked to attend at 8:00–10:00 am in the same room with constant temperature and humidity to minimize diurnal variation. Eating/chewing, drinking and brushing teeth were refrained 90 min prior to each appointment. After resting for 10 min, unstimulated whole saliva (UWS) was collected by a sterile and pre-weighed plastic container every 30 s for a 5 min period. Stimulated whole saliva (SWS) was collected by asking patients to chew a piece of sterilized silicone rubber tubing (4*8 mm) and to spit all the saliva into another container as the same frequency and period of UWS. UWS and SWS were determined by the result of reweighting each container after collection subtracting the weight of empty container^14^.

#### Schirmer I test

We introduced the Schirmer I test to measure lacrimal flow (LF) without previous topical anesthetic to measure the tear secretion. A Whatman special filter paper strip of 20 mm*5 mm (Cytiva®, Shanghai, China) were placed in the lateral 1/3 of the lower bilateral eyelid. The length of the moistened portion of the strip was measured at the fifth minute^15^.

#### ESSDAI and ESSPRI^16^

Disease activity was assessed using and European League Against Rheumatism Sjögren’s Syndrome Disease Activity Index (ESSDAI). These questionnaires were filled out by the patients at baseline and the follow-ups. The ESSDAI includes 12 domains, including cutaneous, respiratory, kidney, articular, muscular, peripheral nervous system, central nervous system, hematological, glandular, constitutional, lymphadenopathic, and immunological, with a total score ranging from 0–3 points.

European Alliance of Associations for Rheumatology SS Patient Reported Index (ESSPRI) is a patient-administered questionnaire to assess disease symptoms on a 10-point scale for pain, fatigue and dryness. The patients were required to show the maximal scale they had experienced during the last 3 days on the VAS ruler. The scores on the ruler were at least 10 cm with 10 visual analogue scales, indicating dryness, pain, and fatigue.

#### Immunological indexes

Patients’ venous blood samples were drawn at 6:00–8:00 am prior to administration of ADSCs injection and at each follow-up. Serum samples were transported at − 20 °C and kept at − 80 °C in a freezer until analysis. Immunological indexes, including IgA, IgG, IgM, complement 3 (C3), complement 4 (C4) and erythrocyte sedimentation rate (ESR), were analyzed using a flow cytometer (Beckman Coulter®, Fullerton, CA).

### Safety assessments

To evaluate safety and toxic effects, clinical laboratory values, vital sign, physical examination and 12-lead electrocardiography were recorded at baseline and follow-ups. A treatment-emergent adverse events (AE) was defined as any untoward symptom, occurred from the first dose of medication until the end of the study. Those were possibly, probably or definitely related to the study medication in the judgment of the investigator.

### Statistical analysis

In this study, data were statistically analyzed using SPSS v20.0 software (IBM Corp, Armonk, NY, USA) and visualized using GraphPad Prism 7 (GraphPad Software Inc., La Jolla, CA, USA). The demographic and health status information at baseline was summarized using Means (Standard Deviation, SD) for continuous variables and Frequencies (Percentages) for categorical variables. Data were first examined for normality by the Kolmogorov–Smirnov test and, the data that achieved normality were analyzed using parametric methods while the others used non-parametric methods. Differences in continuous variables between groups were tested by t-test or MannWhitney U test, while differences in categorical variables were tested by Chi-squared tests or Fisher’s exact tests. In our trial, Unpaired t-test was applied to test UWS, SWS, LF, ESSDAI and ESSPRI, immunological indexes to determine significant difference between baseline and follow-up. Paired t-test was used to analyze significant differences of intergroup at each data point. All reported p-values were from two-side tests and compared with a significance level of 0.05 (*α* = 0.05).

## Results

**Figure 1:**
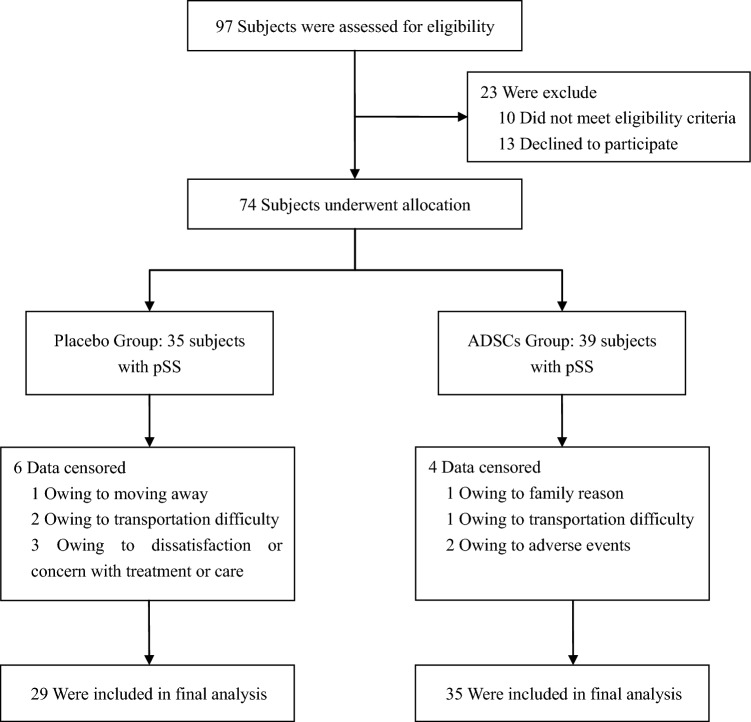
Flow diagram of this clinical trial.

This study is a 6-month randomized, triple-blind, placebo-controlled clinical trial. A total of 97 subjects were screened for this trial from August 2021 to September 2022. 74 subjects who met the including criteria were allocated in two groups. With 10 subjects were withdrawn from the trial, a total of 64 subjects (94.1%, aged from 35 to 74 years, 16 male and 48 female) finally completed the treatments and the follow-up assessments (Fig. 1). The demographic and health status information at baseline are outlined in Table 1. At the same time, clinical evaluation was performed and disease history of patients with pSS was recorded (Table 2). The baseline characteristics did not differ significantly between Placebo and ADSCs Groups (*P* > 0.05).

**Table 1:** The demographic and health status information at baseline.

	Placebo	ADSCs	*t/χ*^*2*^	*P*
Age, mean (SD), y	57.6 (8.3)	59.1 (11.6)	*t* = − 0.57	0.57
Men, No. (%)	6 (20.7)	10 (28.6)	*χ*^*2*^ = 0.43	0.84
Han nationality, No. (%)	27 (93.1)	30 (85.7)	*χ*^*2*^ = 0.89	0.35
Body weight, (SD), kg	57.0 (6.9)	55.6 (9.0)	*t* = 0.71	0.48
BMI, mean (SD)^a^	23.9 (6.0)	22.2 (4.9)	*t* = 1.18	0.23
Blood pressure, mean (SD)				
SBP (mmHg)	125.1 (23.2)	116.1 (27.1)	*t* = 1.41	0.16
DBP (mmHg)	73.2 (13.6)	67.3 (15.0)	*t* = 1.63	0.11
Marital status, No. (%)				
Married or cohabiting	21 (79.3)	24 (68.6)	*χ*^*2*^ = 0.37	0.95
Divorced	4 (13.8)	6 (17.1)		
Widowed	1 (3.4)	2 (5.7)		
Single	3 (10.3)	3 (8.6)		
Education status, No. (%)^b^				
Incomplete secondary education	10 (34.5)	12 (34.3)	*χ*^*2*^ = 1.24	0.74
Ccomplete secondary education	15 (51.7)	19 (54.3)		
Complete university education	3 (10.3)	4 (11.4)		
Postgraduate	1 (3.5)	0 (0.0)		
Economic status, No. (%)				
Low-income (< 100$/y )	5 (17.2)	8 (22.9)	*χ*^*2*^ = 0.55	0.76
Middle-income ( 1000–1000$/y )	23 (79.3)	25 (71.4)		
High income (> 10,000$/y )	1 (3.5)	2 (5.7)		

^a^BMI, body mass index (calculated as weight in kilograms divided by height in meters squared).

^b^According to the per capita income standard of the World Bank (2020 version).

There is no significant of demographic and health status information in the two groups at baseline.

**Table 2:** Clinical evaluation and history of patients with pSS.

	Placebo	ADSCs	*t/χ*^*2*^	*P*
Age of diagnosis, mean (SD), y	53.2( 9.8)	58.4 (11.6)	*t* = 1.91	0.06
Symptom duration before Diagnosis, mean (SD), y	3.5 (2.0)	3.4 (1.8)	*t* = − 1.91	0.97
UWS, mean (SD), ml	2.1 (0.7)	1.8 (0.6)	*t* = 1.85	0.07
SWS, mean (SD), ml	3.4 (0.8)	3.5 (0.9)	*t* = − 0.45	0.64
LF, mean (SD), cm	4.1 (1.1)	3.9 (1.4)	*t* = 0.63	0.53
Positive salivary gland biopsy (focus score ≥ 1), No. (%)	27 (93.1)	32 (91.4)	*χ*^*2*^ = 0.06	1.00
Autoantibodies				
Anti-SSA, No. (%)	22 (75.9)	23 (65.7)	*χ*^*2*^ = 0.78	0.42
Anti-SSB, No. (%)	19 (65.5)	20 (57.1)	*χ*^*2*^ = 0.47	0.61
Current systemic involvement, No. (%)	8 (27.6)	11 (31.4)	*χ*^*2*^ = 0.11	0.79
Disease activity indexes				
ESSDAI, No. (%)	19.2 (2.5)	17.1 (1.9)	*t* = 3.82	0.00
ESSPRI, No. (%)	4.9 (1.2)	4.5 (1.1)	*t* = 1.74	0.15

**Figure 2:**
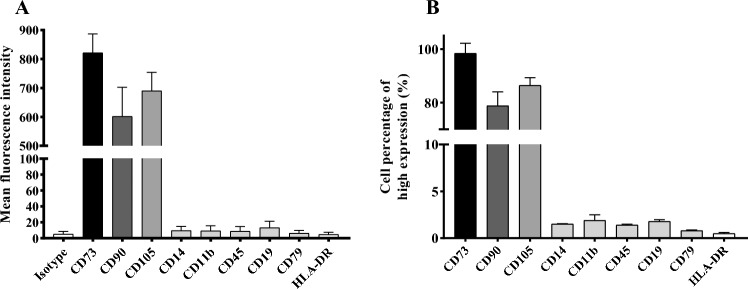
The expression of mesenchymal stem cell markers with flow cytometry analysis. (**A**) Mean fluorescence intensity (MFI) of specimen before clinical injection. (**B**) Cell percentage of high expression after statistical analysis. A high expression of CD73 (98.4% ± 5.2), CD90 (78.8% ± 5.2) and CD105 (86.4% ± 2.9 ) were observed, and CD14 (1.5% ± 0.04), CD11b (1.9% ± 0.6), CD45 (1.4% ± 0.08), CD19 (1.8% ± 0.18) CD79 (0.8% ± 0.07), and HLA-DR (0.5% ± 0.1) express scarcely.

The expression of mesenchymal stem cell markers were confirmed by flow cytometry analysis, including CD73 (98.4% ± 3.8), CD90 (78.8% ± 5.2) and CD105 (86.4% ± 2.9). At the same time, CD14 (1.5% ± 0.04), CD11b(1.9% ± 0.6), CD45 (1.4% ± 0.08), CD19 (1.8% ± 0.18), CD79 (0.8% ± 0.07), and HLA-DR (0.5% ± 0.1) express scarcely (Fig. 2).

**Figure 3:**
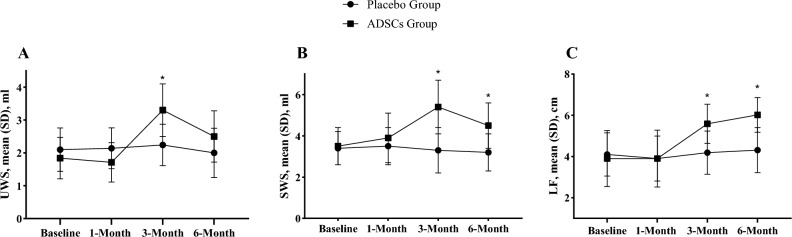
Clinical examination at baseline and each follow-ups in the two groups. *Significantly enhanced ADSCs group compared to placebo group. UWS: unstimulated whole saliva; Stimulated whole saliva; LF: lacrimal flow.

At the clinical examination, baseline and 1-month comparison between the two groups revealed no significant difference in Sialometry and Schirmer I test (P > 0.05). However, in 3-month, a significant difference in UWS and SWS was observed between the two groups (*P* < 0.05). Moreover, the UWS in ADSCs Group decreased and failed to reveal a significant differences when compared to Placebo Group in 6-month (*P* > 0.05). The mean strip wetness of lacrima and LF was significantly higher in the ADSCs groups (*P* < 0.05).A possibility is that there could have a longer duration of therapeutic effects with ADSCs for lacrimal gland (Fig. 3).

**Figure 4:**
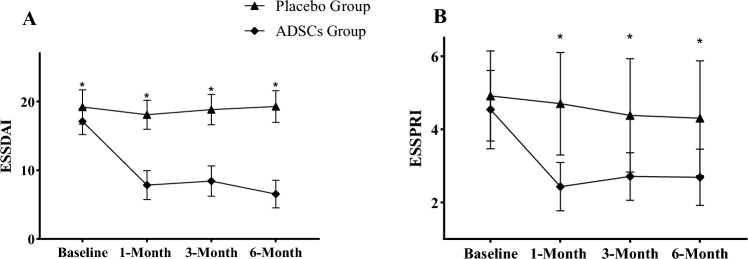
(**A**, **B**)Disease activity assessment at baseline and each follow-ups in the two groups. * Significantly reduction in ADSCs Groups compared to Placebo Group; ESSDAI: European League Against Rheumatism Sjögren’s Syndrome Disease Activity Index; ESSPRI: European Alliance of Associations for Rheumatology SS patient Reported Index.

Baseline comparison of the two groups revealed a significant difference in ESSDAI. Nevertheless, we also found a great improvement with intergroup comparison from baseline to follow-up (*P* < 0.05). In this trial, articular, cutaneous and muscular domains mostly contributed to the decrease of ESSDAI score. Meanwhile, when comparing the outcome of ESSPRI, there is a significant difference between the two groups in the follow- ups(*P* < 0.05) (Fig. 4).

Additionally, the changes of laboratory tests of immunological factors are presented in Table 3. The IgA level did not differ significantly at all time points (*P* > 0.05). There were abatement of IgG, IgM, C3, C4 and ESR between the two groups(*P* < 0.05). Notably, not all the changes reached a statistical significance in each time point.

**Table 3:** The changes of immunological indexes.

	Placebo	ADSCs	*t*	*P*
IgA, mean (SD), g/L (normal range, 0.71–3.85)				
Baseline	2.7 (0.8)	2.6 (0.5)	0.6	0.54
1-month	2.6 (0.8)	2.9 (0.5)	− 1.7	0.08
3-month	3.2(1.0)	2.9 (0.6)	1.6	0.10
6-month	3.0 (0.9)	3.0 (0.7)	-0.3	0.77
IgG, mean (SD), g/L (normal range, 7–16.6)				
Baseline	17.4 (4.7)	18.8 (4.0)	− 1.4	0.18
1-month	16.9 (5.3)	15.3 (3.8)	− 1.4	0.19
3-month	16.9 (5.1)	13.4 (3.1)	3.3	0.00*
6-month	17.7 (3.3)	14.4 (3.5)	3.9	0.00*
IgM, mean (SD), g/L (normal range, 0.7–1.7)				
Baseline	1.7 (0.5)	1.7 (0.6)	0.4	0.72
1-month	1.7 (0.7)	1.4 (0.6)	1.7	0.09
3-month	1.8 (0.6)	1.3 (0.5)	3.4	0.00*
6-month	1.9 (0.7)	1.3 (0.5)	3.7	0.00*
C3, mean (SD), g/L (normal range, 0.70–1.28)				
Baseline	1.4 (0.3)	1.5 (0.4)	− 1.0	0.33
1-month	1.6 (0.4)	1.2 (0.5)	3.5	0.00*
3-month	1.4 (0.4)	1.0 (0.5)	3.6	0.00*
6-month	1.3 (1.0)	1.1 (0.8)	2.6	0.01*
C4, mean (SD), g/L (normal range,0.2–0.6)				
Baseline	0.44 (0.08)	0.50 (0.12)	− 0.9	0.45
1-month	0.41 (0.10)	0.24 (0.09)	2.9	0.00*
3-month	0.38 (0.12)	0.28 (0.11)	1.7	0.02*
6-month	0.49 (0.15)	0.31 (0.13)	1.9	0.01*
ESR, mean (SD), mm/h (normal range, 0–15)				
Baseline	12.5 (0.9)	11.9 (1.2)	0.8	0.38
1-month	12.8 (0.8)	5.3 (0.8)	4.2	0.00*
3-month	11.4 (1.4)	4.8 (1.1)	3.4	0.00*
6-month	12.1 (1.0)	5.4 (1.5)	4.0	0.00*

## Discussion

This is a triple-blinded clinical trial aiming at the effect of ADSCs treatment on pSS patients. In general, the results of our study indicate that ADSCs improved the symptoms in to a certain extend objectively and subjectively. The dysfunction of salivary and lachrymal glands was significantly improved in a short period. The level of immunological and inflammatory indexes was reduced after ADSCs treatment. Previous studies found that transplantation of ADSCs could significantly inhibit autoimmune progression in MRL/lpr mice by injecting with ADSCs via the tail vein^17^. In agreement with our results, Sun et al. suggested that mesenchymal stem cell (MSC) transplantation is an effective treatment for pSS to improve salivary gland pathology and exocrine function. They provide a novel mechanism of MSC treatment and transplantation of ADSCs could significantly inhibits autoimmune progression indicating a brand-new perspective of MSC therapy in SS^18^.

Although the past few years have led to much greater knowledge of the features of patients with pSS, in contrast with other autoimmune diseases such as systemic lupus erythematosus, there is little diagnostic information on clinical pSS^19^. Therefore, the definition of inclusion criteria is a key issue for future clinical trials in this disease. At present, ACR criteria including more objective findings than subjective symptom-based items, firstly introduced by Shiboski at the International Symposium on SS in Bergen in May 2015, is widely used to diagnose pSS^20^. Based on the criteria, two tools for assessing pSS activity and patients-reported outcomes were created, showing a superior performance in responsiveness and discrimination compared to other patient-administered questionnaires to assess patients’ symptoms^17,21^.

Because the pathogenesis of pSS is not fully elucidated and has a very heterogeneous course, which made the treatments challenging in the past^22,23^. Artificial tears or oral sprays has been introduced in local treatments, but the effects are limited because they can not modify the evolution of the disease^24^. Systemic of cholinergic parasympathomimetic agonist such as Pilocarpine and Cevimeline has a particular effect on stimulation of tears and saliva. Although the effectiveness and favorable safety have been confirmed in several RCTs, the tolerability and discontinuation rate during the treatment needs further evaluation^25,26^. Several biologic disease modifying antirheumatic drugs have shown promising outcomes in pSS. However, whether patients with biologic medicines have an increased risk for development of malignancies is still under discussion.

Recently, the positive effect of ADSCs therapy has been suggested in autonomous immunity diseases^27^. Sara et al. reported that the ADSCs could be successfully culture adapted and their immunobiological regulation function remains intact in vitro. They did not find any significant functional differences between pSS and ADSCs, suggesting that ADSCs were likely to directly facilitate the autoimmune regulation^28^. In accordance, a clinical trial by Julien et al. showed an improvement intravenous ADSCs therapy in knee osteoarthritis without detecting toxicity, infusional or allergic reactions during the follow-ups^26,29^.

Notably, the origin of ADSCs contributes to the properties of it, such as downstream application. Thus, a standardized protocol for clinical utility will be helpful. A controlled trial by Vangsness et al. introduced a dose of 3 million cells for an individual for an injection to the knee following partial medial meniscectomy and showed an improvement of recovery^30^. Lamo-Espinosa reported that they set different groups of cell dosage (5 × 10^4^, 10 × 10^4^ and 20 × 10^4^ cells per kilogram) and the results revealed that more MSCs did not lead to better effects^31^. In our previous study and clinical practice, we also assessed the effective and safe dose of administration. Based on the abovementioned finding and our previous study, we set 5 × 10^4^ cells per kilogram/day and injection frequency as the greatest therapeutic effects in this trial.

Another finding from our trial is that there were no other adverse events except for two patients with itching sensation in the skin lasting no more than 2 h. A possible reason may be the method used for ADSCs expansion and culture in our study. Unlike in other studies, we used human platelet lysate as the culture media that may have the advantage of diminishing the immune reaction of animal-derived factors. Until recently, fetal bovine serum (FBS) was the supplement of choice for growing MSCs. However, there were some concerns that an immune response could be triggered in the host for FBS as being xenogeneic. Abundant growth factors and cytokines in platelets are vital for the growth of any animal cell including stem cells. Additionally, ADSCs are found in numerous human tissues, which could be accessed and obtained easily on the superficial layers of the skin. These make the ADSCs therapy is a promising method in pSS.

Many studies have been conducted to explore the unclear pathogenesis of pSS. Various cells and cytokines such as B cells and T cells are involved in pSS^32,33^. Over recent decades, the crucial role of both innate andadaptive immune mechanisms in the generation of immunopathological pSS lesions has been convincingly demonstrated. Therefore, targeting these cells and their cytokines may help to modulate the immune response of pSS. There is accumulating evidence that mesenchymal stem cell-mediated may induce an optimal balance in the immune system that may lead to a sustained response^34^. A research performed a randomized and controlled study, including 51 patients revealed a significant change concerning α-fodrin IgG and IgM^35^. According to another recent study, IgG, IgA, C3 and C4 were included and there is a significant variety in primary and secondary endpoints at 6-month^36^. In this trial, like in other studies, the results of the present study confirmed that serum IgG, IgM, C3 and C4 significantly decreased in ADSCs Group.

In order to evaluate the efficacy more comprehensively, we chose both ESSDAI and ESSDAI as the index of subjective symptoms and systemic characteristics that showing to be valid and sensitive measures^37^. Furthermore, it is interesting to underline that, in the present study, the variety immunological indexes were accordant with subjective indexes^38^. The association between them could be explained inflammatory reaction play a leading role in disease mechanism. That is to say, ADSCs can improve systemic and subjective symptoms by regulation effects on the proliferation of lymphocytes to some extent. In this regard, some evidences have indicated that ADSCs led to alleviate overwhelming systemic inflammatory reaction and organ damage after SS^39^.

A strength of this trial is the use of very strict inclusion criteria and defining activity levels that are crucial for clinical trial design in pSS. The availability of repeated clinical and laboratory assessment over a long follow-up period made it possible to detect the changes of the treatment outcome.

However, there are some limitations to the present trial that need to be addressed. The sample size limited the precision of detection some potential significant differences that we hypothesized to detect prior to the trial. Moreover, further analysis of the ADSCs could be conducted such as assessing the immunomodulatory properties, examining whether cells are present at the injection site and monitoring changes in growth factors or cytokines secreted by MSCs. Further limitation is that the dosage and interval of ADSCs administration have not been thoroughly compared.

## Conclusion

The ADSCs therapy can provide relief of oral and eye’s dryness in our trial in a short time and has potential improvement of subjective and systemic syndromes of pSS.
